# Prise en charge des syndromes myélodysplasiques au Maroc à propos d´une étude mono-centrique

**DOI:** 10.11604/pamj.2020.37.300.20972

**Published:** 2020-12-02

**Authors:** Hicham El Maaroufi, Mounir Ababou, Adnane Hammani, Siham Ahchouch, Salim Jennane, Mehdi Mahtat, Mohamed Mikdmae, Nezha Messaoudi, Kamal Doghmi

**Affiliations:** 1 Service d´Hématologie Clinique, Hôpital Militaire d´Instruction Mohammed V, Rabat, Maroc,; 2 Laboratoire d´Hématologie, Hôpital Militaire d´Instruction Mohammed V, Rabat, Maroc

**Keywords:** Syndrome myélodysplasique, Maroc, étude, Myelodysplastic syndrome, Morocco, study

## Abstract

Il s´agit d´une étude rétrospective de type descriptive et analytique, réalisée au sein du service de l´hématologie clinique de l´hôpital militaire d´instruction Mohammed V de Rabat, étalée sur 10 ans et portant sur 76 patients dont le diagnostic de syndromes myélodysplasiques (SMD) a été posé entre 2008 et 2018. Le nombre total des malades que nous avons recruté est de 76 patients, avec une moyenne annuelle de 7,6 cas. Parmi les 76 patients on dénombrait 57% d´hommes et 43% de femmes. L´âge moyen de notre population est de 65,75 ans ± 12,55. La moyenne d´âge est de 66,88 ± 13,10. Aucun cas de profession exposante n´a été trouvé. 97,3% ont un SMD primaire et seulement 2 patients soit 2,7% ont un SMD secondaire à une chimiothérapie anticancéreuse. Le délai entre la première consultation et le diagnostic du SMD est en moyenne est de 33,6 jours ± 51 avec une médiane de 19 jours. Le scorer pronostique IPSS était de faible risque dans 37,4% des cas, de risque intermédiaire 1 dans 46,6% des cas, risque intermédiaire dans 12% et de risque élève dans 4%. Donc 84% des patients avaient un SMD de faible risque et 16% avaient un SMD de haut risque. Le suivi régulier de nos malades, a permis de déceler de nombreuses complications à type: complications hémorragiques chez 13% des malades, complications infectieuses chez 8% des cas, l´hémochromatose secondaire, conséquence des transfusions itératives chez 6,6% des patients et la transformation en leucémie aiguë myéloïde chez 2,7% des malades. Dans notre étude, l´abstention était le choix thérapeutique pour 42,1% des patients, la transfusion a été préconisé chez 35,5% des patients: par des culots globulaires dans 70% des cas, par des concentrés plaquettaires dans 40% des cas, les chélateurs de fer chez 25% des patients transfusés et l´EPO a été prescrite chez 27% des patients. L´azacitidine était le choix prescrite pour 18% des malades, 50% avaient un SMD de faible risque et 50% avaient un SMD de haut risque. La greffe de moelle représente le seul moyen thérapeutique curatif des SMD, elle était réalisée chez un seul malade ayant un SMD est de haut risque.

## Abstract (en)

We conducted a retrospective descriptive analytical study in the Department of Clinical Haematology at the Mohammed V Military Training Hospital in Rabat over a period of 10 years. This study included 76 patients diagnosed with myelodysplastic syndrome (MDS) between 2008 and 2018. The average number of cases per year was 7.6. Out of 76 patients, 57% were men and 43% were women. The average age of our study population was 65.75 ± 12.55. The average age was 66.88 ± 13.10. No cases of profession exposed to disease was reported. Ninety-seven point three percent of patients had primary myelodysplastic syndrome and only 2 or 2.7% had myelodysplastic syndrome secondary to chemotherapy. The average time between the first visit and the diagnosis of myelodysplastic syndrome was, on average, 33.6 days ± 51, with a median of 19 days. The IPSS prognostic score was: low risk in 37.4% of cases, intermediate risk 1 in 46.6% of cases, intermediate risk in 12% of cases and high risk in 4% of cases. Thus, 84% of patients had low-risk MDS and 16% had high-risk MDS. Regular monitoring of patients showed many complications such as bleeding in 13% of patients, infections in 8% of cases, secondary hemochromatosis as a result of iterative transfusions in 6.6% of patients and transformation to acute myeloid leukemia in 2.7% of patients. In our study, abstention was the therapeutic choice in 42.1% of patients, transfusion was recommended in 35.5% of patients: red cells in 70% of cases, platelet concentrates in 40% of cases, iron chelators in 25% of transfused patients and EPO in 27% of patients. azacitidine was prescribed in 18% of patients, 50% had low-risk MDS and 50% had high-risk MDS. Bone marrow transplant was the only curative treatment for MDS. It was performed in a single patient with high risk MDS.

## Introduction

Les syndromes myélodysplasiques (SMD) englobe toute une gamme hétérogène d´affections chroniques et clonales touchant les cellules souches multipotentes ou bien myéloïdes. Ils se caractérisent par une prolifération anormale des précurseurs médullaires qui présentent un taux d´apoptose accrue, qui aboutit à une hématopoïèse inefficace, responsable de cytopénies périphériques contrastant avec une moelle de richesse généralement conservée ou hyperplasique [1]. Ils sont appelés également les états pré-leucémique vu le risque de progression vers une leucémie aiguë myéloïde (LAM) dans le tiers des cas [1]. Les syndromes myélodysplasiques forment un groupe d´hémopathies myéloïdes clonales généralement acquises, rarement congénitales, qui touchent le sujet âgé avec un âge moyen de survenue de 60 à 70 ans et une légère prédominance masculine [2]. Par ailleurs, des cas de plus en plus jeunes ont été rapportés dans la littérature. Ils sont dotés d´une présentation clinique très hétérogène, le syndrome anémique représente l´élément clinique dominant, mais la découverte se fait d´une manière fortuite dans la moitié des cas à l´occasion d´un bilan de contrôle ou pour explorer une autre affection [2].

Leur diagnostic positif est difficile et nécessite parfois beaucoup de temps et une exploration biologique profonde. Il se base essentiellement sur l´analyse quantitative et qualitative du sang et de la moelle qui objective des signes de dysplasie touchant une ou plusieurs lignées myéloïdes avec moelle souvent riche [2]. En effet, Il est nécessaire d´évaluer le pronostic des patients avant la prise d´une décision thérapeutique, Pour cela plusieurs scores pronostiques ont été proposés, notamment l´IPSS (international prognostic scoring system), IPSS révisé et WPSS (WHO-based prognostic scoring system). Ces scores permettent de diviser les SMD en 2 catégories: le SMD de faible risque et le SMD de haut risque. Pour les sujets atteints d´un SMD de faible risque, la prise en charge repose sur la surveillance de l´évolution de la maladie et la correction des cytopénies par un traitement essentiellement symptomatique (transfusions, chélateurs de fer, antibiothérapie…), afin d´améliorer la qualité de vie des patients. En revanche, la majorité des SMD de haut risque justifient un traitement spécifique, qui va cibler le contrôle de l´évolution leucémique par les agents hypométhylants ou des thérapeutiques plus intensives, mais qu´après une évaluation des comorbidités et de la fragilité du patient [3].

L´allogreffe des cellules souches hématopoïétiques représente le meilleur outil thérapeutique, potentiellement curatif. Mais Elle implique l´existence d´un donneur, un âge généralement inférieur à 65-70 ans, l´absence de comorbidités majeures et un SMD de haut risque pour que le bénéfice ne soit pas contrebalancé par la toxicité [4]. L´évolution est variable: elle peut être progressive avec augmentation des cytopénies et/ou de la blastose médullaire ou assez brutale avec transformation en quelques semaines d´un SMD relativement stable en LAM. La survie est aussi variable selon la catégorie de risque initiale. Les principales causes de décès sont les infections et les hémorragies (60%), de même que la transformation en LAM (30%) [4].

## Méthodes

Il s´agit d´une étude rétrospective de type descriptive et analytique, réalisée au sein du service de l´hématologie clinique de l´hôpital militaire d´instruction Mohammed V de Rabat, étalée sur 10 ans et portant sur 76 patients dont le diagnostic de SMD a été posé entre 2008 et 2018.

**Les patients inclus**: 1) patients des 2 sexes; 2) dont l´âge était supérieur à 18 ans; 3) ayant consulté ou été hospitalisé au service de l´hématologie clinique de hôpital militaire d´instruction Mohammed V de Rabat; 4) entre 2008 et 2018.

**Les patients exclus**: 1) les patients présentant une dysmyélopoïèse secondaire à une carence en vitamine B12.

Le recueil des informations s´est effectué sur des fiches d´exploitation préétablies, de façons passives en étudiant profondément les dossiers médicaux. Cette fiche nous a permis d´étudier: l´aspect épidémiologique, la présentation clinique (antécédents, présentation de la maladie, examen clinique…), le bilan para-clinique.

**Nous avons classé nos malades selon 2 classifications**: 1) classification de l´organisation mondiale de la santé: OMS (l´ancienne 2008 et la nouvelle 2016); 2) classification Franco-Américano-Britannique (FAB).

**Les scores pronostiques**: trois scores ont été calculés: 1) le score IPSS; 2) le score IPSS-R; 3) WPSS.

**La prise en charge thérapeutique, l´évolution**: c´est la surveillance clinico-biologique qui nous a permis de préciser: 1) la réponse aux traitements; 2) la présence d´effets secondaires; 3) les complications; 4) la transformation en leucémie aiguë; 5) les patients perdus de vue; 6) les patients décédés.

**L´analyse statistique**: l´analyse statistique est effectuée en utilisant le logiciel SPSS (version 13.0). Nous avons effectué une analyse descriptive des caractéristiques épidémiologiques, cliniques, biologiques, thérapeutiques et évolutives des patients. En ce qui concerne les variables quantitatives, nous avons calculé les moyennes et écarts-type, minimum et maximum et pour les variables qualitatives les pourcentages. Puis, une analyse univariée a été faite pour comparer les résultats SMD de haut risque et de faible risque. Pour la comparaison, nous avons utilisé les tests paramétriques classiques (test de Khi^2^, test de Levene et test-t) et les tests non paramétriques (test de Mann-Whitney) selon la nature des variables à comparer. Les graphes ont été faits grâce au Microsoft Excel 2013.

## Résultats

Le nombre total des malades que nous avons recruté est de 76 patients, avec une moyenne annuelle de 7,6 cas. Parmi les 76 patients on dénombrait 43 hommes soit 57% et 33 femmes soit 43%. Nous constatons une légère prédominance masculine avec une sex-ratio de 1,3 hommes pour une femme (Tableau 1). L´âge moyen de notre population est de 65,75 ans ± 12,55(34-99) avec des extrêmes allant de 34 à 99 ans. Nous constatons que le SMD touche préférentiellement les patients âgés entre 60 et 79 ans, mais l´atteinte du sujet jeune n´est pas négligeable (3%). La moyenne d´âge est de 66,88 ± 13,10 chez l´homme et de 65,181 ± 12,90 chez la femme. Aucun cas de profession exposante n´a été trouvé. Dans notre série de cas, 74 patients soit 97,3% ont un SMD primaire dont aucun facteur causal n´a pas été retrouvé et seulement 2 patients soit 2,7% ont un SMD secondaire à une chimiothérapie anticancéreuse. Dans 32.8% (nb=29) aucun antécédent (ATCD) n´a été retrouvé, en revanche 67,2% (nb=47) avaient des comorbidités à type de: 1) diabète et ses complications dans 32% (nb=25); 2) HTA dans 29% (nb=22); 3) association HTA et diabète dans 18% (nb=14); 4) maladies auto-immunes (thyroïdite, PR) dans 5,2% (nb=4); 5) cardiopathies 18%. Dans notre série de cas, 29 patients soit 38% n´avaient aucun symptôme en rapport avec la pathologie et le SMD a été découvert de façon fortuite devant l´existence des anomalies biologiques lors d´un bilan systématique.

**Tableau 1:** les différents résultats cliniques, biologiques et pronostiques

Variables	Nombre des patients	Pourcentage (%) des patients
**L´âge (ans)**		
20-39	2	2,6
40-59	18	23,7
60-79	49	64,5
>80	7	9,2
Le sexe		
Homme	43	57
Femme	33	43
**Hémogramme**		
Hb(g/dl)		
>10 g/dl	25	33
8-10 g/dl	21	27
<8 g/dl	12	16
Plaquettes(G/L)		
> 100	25	33
50-100	12	16
< 50	8	10,5
**PNN (éléments/mm^3^)**		
>800	52	68,5
<800	24	31,5
%Blastes médullaires		
<5%	49	64,5
5-10%	16	21
11-20%	8	10,5
>20%	3	4
**Caryotype**		
Favorable	62	82
Intermédiaire	10	14
Défavorable	3	4
**Classification OMS 2016**		
SMD-MLD	43	59
SMD-SLD	1	1,4
SMD-RS	4	5,4
SMD-EB1	15	20,5
SMD-EB2	8	11
SMD- 5q	0	0
SMD-U	0	0
**IPSS**		
Risque faible	28	37,4
Risque inter 2	9	12
Risque élevé	3	4
**IPPS-R**		
Risque très bas	11	14,6
Risque bas	40	53,5
Risque inter	11	14,6
Risque haut	9	12
Risque très haut	4	5,3
**WPSS**		
Risque très bas	0	0
Risque bas	17	22,4
Risque inter	28	36,9
Risque haut	25	32,9
Risque très haut	1	1,3

**Les manifestations cliniques**: le syndrome anémique associant une dyspnée d´effort, pâleur cutanéomuqueuse et une asthénie, représente l´élément clinique dominant, il est retrouvé chez 43 patients soit 56,6%. Ensuite vient le syndrome hémorragique fait d´ecchymoses, gingivorragies ou épistaxis, présent dans 10,5% des cas (nb=8), puis le syndrome infectieux dans 2,6% (nb=2). Le syndrome tumoral reste le moins fréquent.

**Le délai de diagnostic**: c´est Le délai entre la première consultation et le diagnostic du SMD, sa moyenne est de 33,6 jours ± 51 avec une médiane de 19 jours.

### Résultats biologiques

#### Hémogramme

**Cytopénies (type -nb)**: l´anémie représente la cytopénie la plus fréquente dans notre série de cas, elle était présente chez 76% des patients (nb=58), suivie de la thrombopénie retrouvée dans 59% (nb=45) et enfin la leucopénie dans 38% (nb=29). Une cytopénie isolée était retrouvée dans la moitié des cas (nb=38).il s´agit d´une anémie isolée dans 68,4% (nb=26), d´une thrombopénie dans 23,6% (nb= 9), l´atteinte isolée de la lignée blanche est très rare dans 7,8% (nb=3). La bicytopénie était présente dans 29% des cas, et pour la pancytopénie dans 21%. Dans notre série de cas, 76% des patients avaient une anémie (nb=58). Parmi ces 58 malades: 1)26 malades avaient une anémie associée à une autre cytopénie. 2) 22 malades avaient une anémie isolée. Le taux d´hémoglobine moyen est de 10.33 ± 2,22, avec des extrêmes allant de 5,6 à 16,2 et une médiane de 10,05. Nous constatons une nette prédominance de l´anémie normocytaire (73%), suivi de l´anémie macrocytaire et microcytaire. 90% des anémies étaient normochromes avec une Concentration Corpusculaire Moyenne en Hémoglobine (CCMH) comprise entre 32 et 38, seulement 10% qui sont hypochromes. 59% des patients avaient une thrombopénie (nb=45) avec taux moyen de plaquettes de 127G /l.

**Parmi ces 49 patients**: 1) 36 patients avaient une thrombopénie associée à une autre cytopénie; 2) 9 patients avaient une thrombopénie isolée. 38.2% des patients avaient une leucopénie (nb=28) avec un taux moyen des globules blancs de 5129/mm^3^. Vingt d´entre eux présentaient une neutropénie de sévérité variable.

**Parmi ces 28 patients**: 1) 25 patients avaient une leucopénie associée à une autre cytopénie; 2) et seulement 3 malades avaient une leucopénie isolée.

**Le myélogramme**: dans plus de 95% la moelle osseuse était hypercellulaire ou normocellulaire et seulement dans 3% ont une moelle pauvre. Nous avons constaté que dans la majorité des cas la dysplasie est multilignée 96%, seulement 4% des malades présentent une dysplasie unilignée. La dysérythropoïèse est retrouvée dans 81,2% des cas suivie par la dysgranulopoïèse dans 69,5% des cas et de la dysmégacaryopoïèse. La blastose médullaire était présente chez 74 patients soit 97%, le taux de blastes médullaires moyen était de 5,97% avec une médiane de 3%. 64,5% des patients avaient un taux de blaste inférieur à 5%, 21% avaient un taux compris entre 5 et 10, et que 13% avaient un taux supérieur à 10%. Elle est dite positive lorsque le taux de sideroblastes en couronne est supérieur à 15%. Dans notre étude seulement 5,2% (nb=4) des malades ont une coloration de Perls positive.

**L´étude cytogénétique**: le caryotype a été réalisé chez 75 patients (98,7%), 75% avaient un caryotype normal (nb=57) et 25% avaient un caryotype anormal (nb=18). 1) 82% des patients avaient un caryotype favorable (nb=62); 2) 14% des patients avaient un caryotype intermédiaire (nb=10); 3) et seulement 4% avaient un caryotype est défavorable (nb=3).

### Classifications

**Classification Franco-Américano-Britannique (FAB)**: à partir des données morphologiques, la classification FAB a permis de classer les SMD en 4 classes; 1) la plus retrouvée était l´anémie réfractaire avec une fréquence de 60,4% (nb=44); 2) l'anémie réfractaire avec excès de blastes (AREB) représente 31,5% des cas (nb=23); 3) l´anémie réfractaire sidéroblastique idiopathique (ARSI) représente 5,4% des cas (nb=4); 4) la leucémie myélomonocytaire chronique (LMMC) reste la moins fréquente 2,7% (nb=2).

**Classification OMS 2008:** la classification OMS a été réalisée chez 73 patients: 1) la cytopénie réfractaire avec dysplasie multilignée (CRDM) est la catégorie la plus fréquente et représente 59% des cas (nb=43); 2) les AREB représentent 31,5%: a) AREB-1 dans 65%; b) AREB-2dans 35%; 3) ARSI représente 5,4% (nb=4); 4) la cytopénie réfractaire avec dysplasie unilignée (CRDU) représente 1,4% (nb=1); 5) la LMMC représente 2,7%. Même si le caryotype a été réalisé chez 95% des patients, aucun diagnostic de SMD avec syndrome 5q- na été retenu.

**Classification OMS 2016**: la classification OMS subdivise les SMD en plusieurs sous-types en se basant sur les donnes du myélogramme, de la cytogénétique, cette classification met en avant 3 critères majeurs: 1) nombre de lignées dysplasiques: une, deux, ou les trois. 2) pourcentage (%) de sidéroblastes en couronne: significatif si > 15%; 3) pourcentage (%) de blastes.

### Les scores pronostiques

**Le score IPSS**: le score pronostique IPSS a été calculé pour 75 patients soit 98,9%. Le score IPSS est: 1) à 0 chez 37,4% des patients (n=28) ce qui traduit un risque faible; 2) entre 0.5 et 1 chez 46,6 des patients (nb=35) ce qui traduit un risque intermédiaire 1; 3) entre 1.5 et 2 chez 12% des patients (nb=9) ce qui traduit un risque intermédiaire 2; 4) supérieure à 2,5 chez 4% des patients (nb=3) ce qui traduit un risque élevé. Donc 84% de nos patients (nb=63) avaient un SMD de faible risque et 16% (12) un SMD de haut risque.

**Figure 1:**
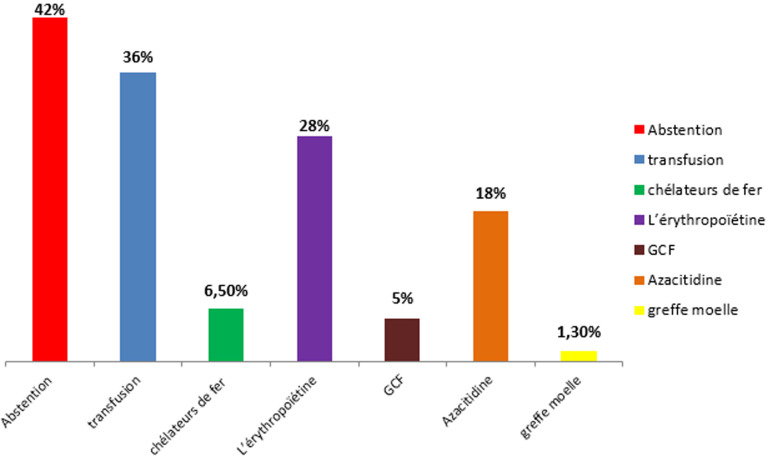
répartition des cas selon le choix thérapeutique

**Le score IPSS-R (Figure 1)**: dans l´IPSS-R le SMD est classé 1) très bas chez 14,6% des patients (nb=11); 2) bas chez 53,5% des patients (nb=40); 3) intermédiaire chez 14,6% des patients (n=11); 4) haut chez 12% des patients (n=9); 5) très haut chez 5,3% des patients (n=4).

**WHO-based Prognostic Scoring System (WPSS):**ce score permet de déterminer la catégorie de risque du SMD et la probabilité que le SMD se transforme en LAM au cours des 5 années suivantes.

**Selon le WPSS**: 1) zéro (0)% avaient un très bas risque (nb=0); 2) 36,9% des patients avaient un bas risque (nb=28); 3) 32,8% des patients avaient un risque intermédiaire (nb=25); 4) 22,4% avaient un haut risque (nb=17); 4)1,3% avaient un très haut risque (nb=1).

**L´évolution et complications (Tableau 2)**. En ce qui concerne l´évolution: 1) 75% des malades sont toujours suivis (nb=57); 2) 7% des malades sont perdus de vue (nb=5); 3)18% des malades sont décèdes (nb=14). La surveillance clinico-biologique et le suivi régulier de nos malades nous a permis de détecter de nombreuses complications chez 30% des malades (nb=23), il s´agit:

**Tableau 2:** l´évolution selon le type du SMD

Variables	Toujours suivis	Perdus de vue	Patients décédés
CRDM	36	2	5
CRDU	1	0	0
AREB 1	10	2	3
AREB 2	4	0	4
ARS	2	1	1
LMMC	1	0	1
SMD 5q	0	0	0
SMD inclassable	0	0	0

**complications hémorragiques**: représentent 43% des complications (nb=10), réparties en: 1) épistaxis chez 3 malades; 2) gingivorragie chez 3 malades; 3) ecchymose chez 2 malades; 4) rectorragies chez 2 malades.

**Complications infectieuses**: qui représentent 26% des cas (nb=6) reparties en: 1) infections pulmonaires chez 3 malades; 2) infections urinaires chez 2 malades; 3) infection ORL chez un seul malade.

**Hémochromatose**: retrouvée chez 22% des complications (nb=5).

**La transformation en leucémie aigüe myéloblastique**: c´est la complication la plus redoutable du SMD, 2 patients sur 76 ont développé une leucémie aiguë (8,7%); 1) le 1^er^ présentait une anémie isolée au moment du diagnostic. La transformation a eu lieu 3 mois après le diagnostic et le décès est survenu presque 1 mois après la transformation leucémique; 2) le 2^e^ présentait une bicytopénie faite d´une anémie et une thrombopénie au moment du diagnostic, la transformation a eu lieu 2 mois après le diagnostic et le décès 2 mois âpres la transformation.

**Le traitement (Tableau 3, Figure 1)**: l´enjeu thérapeutique principal pour les SMD de faible risque est la correction des cytopénies et pour le SMD de haut risque est le contrôle du clone leucémique.

**Tableau 3:** les différents résultats thérapeutiques

Variables	Nombre de malades		
Le choix thérapeutique	SMD de faible risque	SMD de haut risque	P
L´abstention	32	0	0,001
Transfusion	18	9	0,002
EPO	16	5	0,250
G-CSF	2	2	0,570
AZA	7	7	0,001
GCS	0	1	-

**L´abstention**: c´était le choix thérapeutique pour 42,1% des patients porteurs d´un SMD (nb=32). Nous avons trouvé une différence statistiquement significative entre le SMD de faible et de haut risque concernant l´abstention thérapeutique: la moitié des patients porteurs d´un SMD de faible risque n´ayant pris aucun traitement.

**La transfusion**: 35,5% des patients (n=27) avaient un besoin transfusionnel régulier: 1) par des culots globulaires dans 70% des cas (n=19); 2) des concentrés plaquettaires dans 40% des cas (n=11). La fréquence des transfusions étaient de 2,3 culots globulaires par mois pour les patients anémiques et de 6 culots plaquettaires par mois pour les patients ayant une thrombopénie. Nous avons constaté des différences statistiquement significatives en ce qui concerne la transfusion, les patients porteurs d´un SMD de haut risque ont des besoins transfusionnels plus élevés: 75% des patients porteurs d´un SMD de haut risque ont été transfusés (nb=9 sur 12), en revanche seulement 28% des patients ayant un SMD de faible avaient besoins d´une transfusion. (Nb=18 sur 63).

**Les chélateurs de fer**: le recours aux chélateurs de fer a eu lieu chez 25% des patients transfusés (nb=5) 60% parmi eux avaient un SMD de haut risque.

**L´érythropoïétine recombinante**: 27% des patients ont été mis sous l´EPO (nb=21). Même pour l´EPO, Nous n´avons pas trouvé une différence statistiquement significative entre les 2 types du syndrome myélodysplasique: 1) 41% des patients ayant un SMD de haut risque ont été mis sous l´EPO (nb=5 sur 12); 2) en ce qui concerne le SMD de faible risque seulement 25% ont été mis sous l´EPO (nb=16 sur 63).

**Facteur de stimulation des colonies de granulocytes (G-CSF)**: prescrits chez 4 patients soit 5,3% des patients, les différences n´étaient pas statistiquement significatives.

**Azacitidine (AZA):** c´est un moyen thérapeutique qui a été choisi pour 14 patients soit 18%. Les différences en ce qui concerne la prescription de l´AZA entre les 2 types du SMD étaient statistiquement significatives, vu que: 1) 58% des patients porteurs d´un SMD de haut risque avaient besoin de l´AZA (nb= 7 sur 12); 2) et seulement 11% des malades ayant un SMD de faible risque (nb=7 sur 63).

**L´allogreffe de moelle**: le seul patient qui a été greffé dans notre étude est un porteur d´un SMD de haut risque.

## Discussion

Selon notre étude, la fréquence moyenne annuelle des SMD est de 7,6 cas par an (76 cas sur 10 ans). Ce qui rejoint, la série de Matsuda *et al*. [5] réalisée entre 1976 et 1997 au Japon où la fréquence était de 6,5 cas/an (131 cas sur 20ans), celle de la série de Benhassen *et al*. [6] réalisée entre au 2006 et 2010 en Tunisie dont la fréquence était de 10 (40 cas sur 4 ans), les séries algériennes de Bouali *et al*. [7] et de Guezlane *et al*. [8] les fréquences respectives de 8 et 7 cas par an. Par contre, cette fréquence reste inférieure à celle rapportée par l´étude nationale de Yahyaoui *et al*. [9] réalisée entre 2006 et 2015 à Rabat dont la fréquence était de 17 cas/an (155 cas sur 9), celles retrouvées dans les séries européennes: Troussard et al en France [10] ont retrouvés 41 cas par an (703 cas en 17 ans), Navarro *et al*. en Espagne [11] ont retrouvés 28 cas par an (311 cas en 11 ans), Germing *et al*. [12] ont retrouvés 30.8 cas par an (308 cas en 10 ans), Bernasconi *et al*. [13] ont retrouvés 35 cas par an. Durant notre étude, le SMD a été diagnostiqué chez 76 patients parmi eux 43 hommes et 33 femmes, nous constatons une prédominance masculine avec un sexe ratio de 1,3 femmes pour un homme. Nos résultats se rapprochent de ceux rapportés par plusieurs séries. A titre d´exemple, la série de Baiza *et al*. [14] réalisé à Casablanca entre 2008 et 2010, dont le sex-ratio est de 1.31, de Navarro *et al*. [11] en Espagne où le sex-ratio est de 1,44, de Benamor *et al*. [15] en Tunisie, de Matsuda *et al*. [5] au Japon, de Yahyaoui A *et al*. [9] à Rabat, les sex-ratios respectifs étaient de: 1,2; 1,14 et 1,58. Par contre, plusieurs études retrouvent une prédominance féminine franche: l´étude française de Lorand *et al*. en 2006, l´étude de Guezlane *et al*. de même pour l´étude de Bouali [7] *et al*. les sex-ratios respectifs étaient: 0,93; 0,9 et 0,86. Dans notre étude, L´âge moyen était de 65,75 ans ± 12,55, (34; 99) avec des extrêmes allant de 34 à 99 ans. Nos résultats concernant l´âge se rapprochent de ceux rapportés par plusieurs séries: la série de Belakhal *et al*. [16] dont l´âge moyen était de 65 ans, la série nationale de Yahyaoui *et al*. [9] où l´âge moyen était de 62 ans, la série de Benhassen [6] avec un âge moyen de 68 ans. Par contre, nos patients étaient plus jeunes par rapport à ceux de plusieurs séries: la série de Navarro *et al*. [11], la série de Ma Xiami [17] *et al*. la série de Troussard *et al*. [10], la série Germing [12] *et al*. la série de Dinmohammed *et al*. [18], l´âge moyen était respectivement: 74; 76; 74; 72; 74 ans. Ceci peut être expliqué par le vieillissement de la population européenne. D´autres études, ont révélé un âge moyen plus bas: l´étude nationale de Baiza *et al*. [14] où l´âge moyen était de 50,5 ans, l´étude de Bouali [7] *et al*. dont l´âge moyen était de 56,6 ans, l´étude Matsuda *et al*. [5], l´étude d’Ehsan [19] *et al*.au Pakistan et l´étude de Samba *et al*.au Sénégal [20], où l´âge moyen était respectivement de: 57; 46,21; 48,31 ans. Selon notre étude nous constatons que la tranche d´âge la plus touchée est comprise entre 60 et 79 ans, ce qui rejoint la littérature. Le SMD se manifeste à un âge proche que ce soit chez les hommes ou chez les femmes avec des moyennes d´âge respectives de 66,88 ± 13,10 chez l´homme et de 65,181 ± 12,90 chez la femme, ceci concorde avec les résultats de l´étude de Bouali *et al*. [7], l´étude de Samba *et al*. [20]. En revanche, plusieurs études affirment que les femmes manifestent le SMD à un âge plus jeune que les hommes. Notamment, dans la série de Kuendgen *et al*. l´étude de Benamor [15] où l´âge moyen chez les femmes est de 62 ans et de 66 ans chez les hommes, et l´étude de Ndiaye [21] dont l´âge moyen chez les hommes est de 51,56 ans contre 41 ans chez les femmes. Seulement 2,6% de nos patients ont un SMD secondaire à une chimiothérapie anticancéreuse. Ce pourcentage est identique à celui rapporté par l´étude d´Avgerinou *et al*. [22] réalisée en Grèce entre 1990 et 2009. En revanche, il est inférieur à celui retrouvé dans plusieurs études, notamment l´étude de Neunkirchen *et al*. [23] réalisée en Allemagne entre 1996 et 2009 et l´étude de Mukiibi *et al*. [24] réalisée en Afrique centrale en 1994 avec des taux respectifs de SMD secondaire de 15%, 9% et 9,5%. Dans l´étude espagnole d´Iglesia *et al*. [25] aucun cas de SMD secondaire n´a été diagnostiqué. Cette différence pourrait être expliquée par le nombre relativement bas de nos patients. La découverte fortuite d´une anomalie à la numération formule sanguine (NFS) nous a amené au diagnostic de 61% des SMD alors que dans la littérature, la découverte fortuite est observée dans 50%. Durant notre étude, nous avons constaté que l´élément clinique dominant est le syndrome anémique fait d´une asthénie, pâleur cutané muqueuse, vertige, suivi par le syndrome hémorragique et le syndrome infectieux. Cette prédominance de l´anémie est constatée par Najman [26] et Merlat qui la [27] retrouvent dans plus de 90% des cas. Intragumtornchai T *et al*. [28] dans leur étude faite en Thaïlande la retrouvent dans 84,6% des cas. Les comorbidités retrouvées chez nos patients sont dominés par des maladies chroniques souvent observées en gériatrie à savoir le diabète, HTA et les cardiopathies. Ces résultats sont cohérents avec la littérature [29-32].

**Les résultats biologiques. NFS (Tableau 3, Tableau 4)**: l´anémie représente la cytopénie la plus fréquente dans notre série de cas, elle était présente chez 76% des patients, suivie de la thrombopénie retrouvée dans 69% et en fin la leucopénie dans 38%. Une cytopénie isolée est retrouvée dans la moitié des cas. Il s´agit d´une anémie isolée dans 68,4%, d´une thrombopénie dans 23,6%, l´atteinte isolée de la lignée blanche est très rare (7,8%). La bicytopénie était présente dans 29% des cas, et pour la pancytopénie dans 21%. Nos résultats concernant la répartition des cytopénies sont cohérents avec plusieurs études, nous citons parmi elles: l´étude de Baiza *et al*. [14] qui a retrouvée l´anémie chez 96% des malades, la thrombopénie chez 44% et la leucopénie chez 35% des malades. Une cytopénie isolée est retrouvée chez 34% des malades, la bicytopénie chez 23%, et la pancytpénie chez 33% des malades. L´étude de Bernasconi *et al*. [13] rapporte dans 56.6% des cas une anémie, dans 51.3% des cas une thrombopénie et dans 46.4% des cas une leucopénie. La cytopénie isolée est retrouvée dans 41.5% des cas, bicytopénie dans 31.6%, et pancytpénie chez14.7% des malades. L´étude de Massimo [33] *et al*. retrouve l´anémie dans 41% des cas, la thrombopénie dans 31% et la leucopénie dans 36% des cas. L´étude nationale de Yahyaoui *et al*. [9] où la cytopénie isolée était présente dans 57,4% des cas, la bicytopenie dans 24,5% et la pancytopenie 18,1. Le taux moyen d´hémoglobine de nos patients est de 10.33 ± 2,22, avec des extrêmes allant de 5,6 au 16,2 et une médiane de 10,05. Ce taux est supérieur à ceux trouvés par Samba *et al*. [20], Nidaye *et al*. [31], Guezlane *et al*. [8] qui sont respectivement de 4,9g/dl, 4,9g/dl et 6,5g/dl. Selon notre étude, l´anémie était normocytaire chez 73% des patients, macrocytaire chez 22% des patients et microcytaire dans 13% des cas. Cette prédominance du type normocytaire a été déjà notée par Bernard *et al*. [31], Bibi I [34], Hmissi B *et al*. [35] qui la retrouvent respectivement dans 60%, 48% et 53% des cas. Par contre, Samba *et al*. [20], Merlat *et al*. [27] et Lowenthal *et al*. [36] ont retrouvé une prédominance macrocytaire qui est plus fréquente dans la littérature et qui pourrait s´expliquer par le phénomène de « vieillissement » des cellules souches hématopoïétiques, marqué par la perte de télomères chromosomiques occasionnant des accidents de réplication de l´acide désoxyribose nucléique (ADN) avec réduction du nombre de mitoses des précurseurs médullaires qui donnent ainsi naissance à des cellules matures de grande taille.

**Tableau 4:** comparaison de la fréquence globale avec les autres séries de cas

Auteur	Pays	Fréquence
Notre série	Maroc	7,6 cas/an
A yahyaoui *et al*. [9]	Maroc	17 cas/an
Bouali *et al*. [7]	Algérie	8 cas /an
Guezlane *et al*. [8]	Algérie	7cas /an
Benhassen *et al*.[6]	Tunisie	10 cas/an
Navarro *et al*. [11]	Espagne	28 cas /an
Bernasconi *et al*. [13]	Italie	35 cas /an
Troussad *et al*. [10]	France	41 cas /an
Germing *et al*. [12]	Allemagne	30,8 cas /an

**Le Myélogramme**: le myélogramme a été réalisé chez tous les patients et a permis de poser le diagnostic dans plus de 85% en précisant des signes de dysplasie et le pourcentage des blastes. La dysérythropoïèse est retrouvée dans 81,2% des cas suivie par la dysgranulopoïèse dans 69,5% des cas et de la dysmégacaryopoïèse dans 59% des cas. Nos résultats concordent avec l´étude d´Ehsan *et al*. [19] et l´étude de Hmissi *et al*. [36] qui retrouvent respectivement une dysérythropoïèse dans 89% et 86%. Concernant la blastose médullaire, 64,5% de nos patients ont un taux de blaste inférieur à 5%, 21% présentent un taux compris entre 5 et 10, 10,5% ont un taux compris entre 11 et 20 et seulement 4% ont un taux supérieur à 20%. Ces résultats diffèrent de ceux rapportés par: l´étude de Bernasconi *et al*. [13] retrouve dans 58.45% des cas un taux de blastes inférieur à 5%, dans 12.62% des cas un taux blaste entre 5 et 10%, dans 18.94% des cas un taux de blastes entre 11 et 20% et un taux blastes supérieur à 20% dans 9.97% des cas. La série de Massimo *et al*. [33] qui rapporte un taux de blastes inferieur a 5% que dans 41% des cas, un taux compris entre 5 et 10% dans 38,7% des cas, dans 12%, un taux compris entre 11 et 20% dans 12% et dans 8% des cas un taux supérieur à 20%.

**Etude cytogénétique**: le caryotype a été réalisé chez 98,7% des patients, il est revenu normal chez 75% des malades. Les anomalies génétiques trouvées sont à type la trisomie 8, les anomalies du chromosome 7 [monosomie 7, del (7q), la del (20q)] et la perte du Y. Ceci concorde avec la littérature [1,37,38]. Durant notre étude, le caryotype est revenu favorable dans 82% cas, intermédiaire dans 14% et défavorable que dans 4% des cas. Ces résultats se rapprochent à ceux rapportés par l´étude de Greenberg *et al*. [29] ou le caryotype était favorable dans 70%, intermédiaire dans 14% des cas et défavorable dans 16%. L´étude de Kelaidi *et al*. [39] dont le caryotype était favorable dans 72.2%, Intermédiaire dans 14% et défavorable 13,8%. L´étude de Matsuda *et al*. [5] retrouve un caryotype favorable dans 77,5% des cas, intermédiaire dans 14,7% et défavorable dans 7,9 des cas. L´âge médian des patients chez qui le caryotype est anormal est de 65 ans, Ce qui est cohérent avec l´étude de Schanz *et al*. [40] dont l´âge médian est de 69 ans.

**Les classifications**: l´anémie réfractaire représente la catégorie la plus fréquente avec une fréquence de 60,3% (nb=44), suivie de l ´AREB qui représente 31% des cas (nb=23), L´ARS est retrouvée dans 6% des cas (nb=4) et la LMMC reste la moins fréquente 2,7% (nb=2). De nombreuses études ont utilisé la classification FAB, parmi elles: 1) l´étude de Greenberg *et al*. [29] qui rapporte une AR dans 36% des cas, une AREB dans 34% des cas, une ARS dans 15% et une LMMC dans 15% des cas; 2) l´étude de Troussard *et al*. [10] qui retrouve l´AR dans 48,4% des cas, l´AREB dans 25%, l´ARSI dans 11% et la LMMC dans 8% des cas; 3) l´étude de Bernasconi *et al*. [41] qui rapport une AR dans 42,6%, une AREB dans 41,3% et une ARS dans 16,1%; 4) l´étude de Hmissi *et al*. [36] qui retrouve chez 70% des patients une AR, l´AREB dans 14% des cas, l´ARSI dans 9% des cas et la LMMC chez 5% des patients; 5) l´étude de Kelaidi *et al*. [39] ou l´AR représente 31,6%, l´AREB représente 42,3%, ARS 17,6% et la LMMC 8,5% des cas; 6) l´étude de Navarro *et al*. [11] retrouve l´AR dans 38,3% des cas, les AREB dans 27% des cas, ARS dans 23,5% et la LMMC dans 11,3 des cas; 7) l´étude de Mukiibi *et al*. [24] qui retrouve l´AR dans 33% des cas, dans 21,4% une AREB, l´ARSI dans 16,7% et la LMMC dans 11,9% des cas.

**Classification OMS**: dans notre série et suivant la classification OMS, on retrouve parmi 76 diagnostics de SMD: 1) la CRMD chez 59% des cas; 2) la CRUD chez 1,4% des cas; 3) l´AREB chez 31,5% des cas; a) AREB1 dans 20,5%; b) AREB 2 dans 11%; 4) l’ARS chez 5,4% cas.

Plusieurs études ont classé leurs patients selon la classification OMS: 1) l´étude de Yahyaoui *et al*. [9] qui rapporte la cytopénie réfractaire avec dysplasie mltilignée (CRMD) chez 60,6% des patients, la cytopénie réfractaire avec dysplasie unilignée (CRDU) chez 0,6%, l´AREB 1 chez 25,8%, l´AREB 2 chez 9,1%, le syndrome 5 q chez 1,3% et le SMD inclassable chez 2,6% des patients. 2) L´étude de Kelaidi *et al*. [39] qui retrouve une CRMD dans 15,4% des cas, une CRUD dans 18,6%, AREB 1 dans 20,7%, AREB 2 dans 18,3%, l´ARS dans 13,3%, SMD inclassable dans 4,9% et le syndrome 5q dans 4,8% des cas. 3) L´étude de Baiza *et al*. [14] dont la CRDM représente 33% des cas, la CRDU 7% des cas, les AREB 1 18,7% des cas, AREB 2 16.3% des cas, les SMD inclassables 7% des cas, le syndrome 5q 2% des cas, et l´ARS 16% des cas. 4) L´étude de Bernasconi *et al*. [13] où les CRDM représentent 3.7% des cas, les CRDU 24.1% des cas, l´AREB 1 14.2% des cas, l´AREB 2 20.6% des cas, l´ARS 11.2% des cas et les SMD inclassables 1.6% des cas. 5) L´étude de Irons *et al*. [42] où le CRDM représente 68% des cas, la CRDU 9.9% des cas, les AREB 16.3% des cas, les SMD inclassables 4,5% des cas, le syndrome 5q 0.3% des cas, et l´ARS 1,1% des cas.

**Scores pronostiques**: nous avons utilisé chez nos malades les 3 scores pronostiques suivants: IPSS, IPSS-R, WPSS. L’IPSS était de faible risque dans 37,4% des cas, de risque intermédiaire 1 dans 46,6%des cas, risque intermédiaire 2 dans 12% et de risque élève dans 4%. Donc 84% des patients avaient un SMD de faible risque et 16% avaient un SMD de haut risque. Nos résultats concordent avec la littérature, et se rapprochent de plusieurs études: 1) l´étude de Greenberg *et al*. [43] retrouve dans 37% des cas un risque faible, dans 40% des cas un risque intermédiaire 1, dans 16% un risque intermédiaire 2 et dans 7% des cas un risque élève. Donc un de SMD faible risque dans 77% des cas et de haut risque dans 23% cas. 2) L´étude de Massimo *et al*. [33] rapporte un risque faible chez 36% des patients, chez 43% un risque intermédiaire 1, chez 18% un risque intermédiaire 2 et dans 3% des cas un risque élève (79% SMD de faible risque et 21% un SMD de haut risque). 3) L´étude de Schanz *et al*. [44], avec un risque faible chez 29,5% des cas, intermédiaire 1 chez 39.2% des cas, intermédiaire 2 chez 19.4% des cas, haut risque chez 11,3% des cas. 4) L´étude de Bernasconi *et al*. [13] ont trouvés un risque faible dans 23,4% des cas, risque intermédiaire 1 chez 36,4% des cas, risque intermédiaire 2 dans 22% et un risque élève dans 18% des cas (59,8 SMD de faible risque et 41,2% SMD de haut risque). 5) L´étude de Kelaidi *et al*. [39] retrouve un risque faible dans 34,8% des cas, un risque intermédiaire dans 32,5% des cas, un risque intermédiaire dans 16,3% et un risque élevé dans 16,3% des cas (67,3% ont SMD de faible risque et 32,6 ont un SMD de haut risque). En ce qui concerne le score IPSS-R, nous avons trouvé un risque très bas chez 14,6% de nos malades, un risque bas dans 53,3%, un risque intermédiaire chez 14,6%, un risque haut chez 12% et un risque très haut chez 5,3 de nos malades. Ce qui est proche des donnés citées dans la littérature, notamment l´étude de Schanz [44] qui a trouvé un risque très bas chez 19% des cas, bas chez 38% des cas, intermédiaire chez 20% des cas, haut chez 13% des cas, et très haut chez 10% des cas.

**Evolution**: le suivi régulier de nos malades, a permis de déceler de nombreuses complications a type: complications hémorragiques chez 13% des malades, complications infectieuses chez 8% des malades, l´hémochromatose secondaire, conséquence des transfusions itératives chez 6,6% des malades et la transformation en leucémie aiguë myéloïde chez 2,7% des malades. 18,5% de nos malades sont décédés (nb=14), 2 parmi eux par transformation leucémique. Ce taux est inférieur à celui retrouvé par Massimo *et al*. [38], où le pourcentage des malades décédés était de 41% dont 32% par transformation leucémique.

**Traitement**: le choix des traitements et les modalités de la prise en charge thérapeutique des syndromes myélodysplasiques sont en majeure partie déterminés par le score pronostique IPSSS. Dans notre étude, l´abstention était le choix thérapeutique pour 42,1% des patients, la transfusion a été préconisé chez 35,5% des patients (par des culots globulaires dans 70% des cas, par des concentrés plaquettaires dans 40% des cas (n=11)), les chélateurs de fer chez 25% des patients transfusés et l´EPO a été prescrite chez 27% des patients. L´azacitidine était le choix prescrit pour 18% des malades (nb=14), parmi ces 14 malades: 1) 7 avaient un SMD de faible risque; 2) 7 avaient un SMD de haut risque. La greffe de moelle représente le seul moyen thérapeutique curatif des SMD, elle était réalisée chez un seul malade ayant un SMD est de haut risque.

Dans l´étude de Kelaidi *et al*. [39], 61% des patients ont bénéficié de transfusion de culots globulaires et 61,7% ont reçu des culots plaquettaires. L’azacitidine et la décitabine ont été utilisées chez 18% des patients de cette expérience américaine. Dans l´étude de Ben Hassan *et al*. [6], le traitement était symptomatique dans 57% des cas, une seule allogreffe de moelle osseuse et abstention thérapeutique chez 27,5% des patients. En ce qui concerne l´étude de Bernasconi *et al*. [13], la majorité des patients des n´ont bénéficié que d´un support transfusionnel (75,7%).

## Conclusion

Les syndromes myélodysplasiques constituent un ensemble d´hémopathies clonales malignes de la cellule souche hématopoïétique. Vu l´absence de registre national, cette étude constitue un reflet de cette pathologie au Maroc. Les résultats observés rejoignent ceux décrits dans la littérature. La mise en place d´un registre national permettra de mieux identifier les besoins des centres nationaux à la fois dans le diagnostic et aussi dans sa prise en charge en mettant en place un référentiel national adapté au pays.

### Etat des connaissances sur le sujet

- Les syndromes myélodysplasiques constituent un ensemble d´hémopathies clonales malignes de la cellule souche hématopoïétique;
- La littérature nous rapporte peu d´études africaines faites sur ce sujet;
- Il y a une différence dans la prise en charge des différents cas syndromes myélodysplasiques.

### Contribution de notre étude à la connaissance

- Elle est l´une des rares études marocaines sur les patients atteints de syndrome myélodysplasique;
- Elle mesure l´impact des facteurs pronostiques dans la survie des patients;
- L´azacitidine utilisée dans la prise en charge des patients de hauts risques.
